# *In utero* and early life exposure to diesel exhaust air pollution increases adult susceptibility to heart failure in mice

**DOI:** 10.1186/1743-8977-10-59

**Published:** 2013-11-26

**Authors:** Chad S Weldy, Yonggang Liu, Yu-Chi Chang, Ivan O Medvedev, Julie R Fox, Timothy V Larson, Wei-Ming Chien, Michael T Chin

**Affiliations:** 1 Division of Cardiology, Department of Medicine, University of Washington School of Medicine, Seattle, WA, USA; 2 Department of Pathology, University of Washington School of Medicine, Seattle, WA, USA; 3 Department of Environmental and Occupational Health Sciences, University of Washington School of Public Health, Seattle, WA, USA; 4 Center for Cardiovascular Biology, University of Washington School of Medicine, Box 358050850 Republican Street, Room 353, Seattle, WA 98109, USA

**Keywords:** Air pollution, Diesel exhaust, Heart failure, Fetal origins of adult disease, Barker’s hypothesis

## Abstract

**Background:**

Fine particulate air pollution (PM_2.5_) is a global health concern, as exposure to PM_2.5_ has consistently been found to be associated with increased cardiovascular morbidity and mortality. Although adult exposure to traffic related PM_2.5_, which is largely derived from diesel exhaust (DE), has been associated with increased cardiac hypertrophy, there are limited investigations into the potential effect of *in utero* and early life exposure on adult susceptibility to heart disease. In this study, we investigate the effect of *in utero* and early life exposure to DE on adult susceptibility to heart failure.

**Methods:**

Female C57BL/6 J mice were exposed to either filtered air (FA) or DE for 3 weeks (≈300 μg/m^3^ PM_2.5_ for 6 hours/day, 5 days/week) and then introduced to male breeders for timed matings. Female mice were exposed to either FA or DE throughout pregnancy and until offspring were 3 weeks of age. Offspring were then transferred to either FA or DE for an additional 8 weeks of exposure. At 12 weeks of age, male offspring underwent a baseline echocardiographic assessment, followed by a sham or transverse aortic constriction (TAC) surgery to induce pressure overload. Following sacrifice three weeks post surgery, ventricles were processed for histology to assess myocardial fibrosis and individual cardiomyocyte hypertrophy. mRNA from lung tissue was isolated to measure expression of inflammatory cytokines *IL6* and *TNF*α.

**Results:**

We observed that mice exposed to DE during *in utero* and early life development have significantly increased susceptibility to cardiac hypertrophy, systolic failure, myocardial fibrosis, and pulmonary congestion following TAC surgery compared to FA control, or adult DE exposed mice. *In utero* and early life DE exposure also strongly modified the inflammatory cytokine response in the adult lung.

**Conclusions:**

We conclude that exposure to diesel exhaust air pollution during *in utero* and early life development in mice increases adult susceptibility to heart failure. The results of this study may imply that the effects of air pollution on cardiovascular disease in human populations may be strongly mediated through a ‘fetal origins’ of adult disease pathway. Further investigations on this potential pathway of disease are warranted.

## Background

Epidemiological investigations have consistently found that exposure to fine particulate matter air pollution (ambient particles with diameter of <2.5 μm, PM_2.5_) is associated with an increased risk of cardiovascular morbidity and mortality [1]. In the most recent Global Burden of Disease study [2], ambient particulate matter pollution was found to be the 9th cause of disease worldwide, reaching as high as the 4th cause of disease in East Asia. In addition, household air pollution from solid fuel combustion is considered the 4th cause of disease worldwide, and the 1st cause of disease in South Asia [2]. These predictions of relative cause of disease are largely based upon our current understanding of associations between adult PM exposures and cardiovascular disease, where a rich body of epidemiology literature has consistently found strong associations between PM exposure and risk of cardiovascular morbidity and mortality [3], including evidence to suggest exposure to traffic related air pollution accelerates the progression of cardiac hypertrophy [4,5]. Cardiac hypertrophy is a critical indicator of future risk of heart failure, and a recent meta-analysis of air pollution and risk of heart failure corroborates these observations, where risk of heart failure hospitalization and death is positively associated with PM_2.5_ exposure [6].

A large number of laboratory investigations utilizing animal models and controlled exposure facilities have widely proved biological plausibility behind these epidemiological observations where studies have observed that both acute and chronic exposures to PM_2.5_ impair vascular reactivity [7-10], incite vascular inflammation and accelerate the progression of atherosclerosis [11-14], and promote the progression of cardiac hypertrophy as well as systolic and diastolic dysfunction [15,16]. Although there is overwhelming evidence that adult exposure to particulate air pollution has deleterious cardiovascular effects, the effects of *in utero* and early life exposure to PM_2.5_ on adult susceptibility to cardiovascular disease has currently not been investigated.

Work by Barker and colleagues has demonstrated that *in utero* and early life development is a critical window in programming adult susceptibility to cardiovascular disease [17-23]. In particular, impaired fetal growth is associated with adult hypertension and risk of mortality [17]. Recently, there has been a growing consensus that exposure to PM_2.5_ during pregnancy impairs fetal development, resulting in reduced birth weight in human populations [24]. In many urban regions diesel exhaust (DE) is an important component of PM_2.5_[25], and recent work using mouse models has observed that exposure to DE during pregnancy incites fetal inflammation, which has been demonstrated to promote offspring to be susceptible to obesity as well as altered pulmonary responses to subsequent ozone exposure [26,27]. From these previous observations, we hypothesized that exposure to DE during *in utero* and early life development will promote an increase in susceptibility to adult cardiovascular disease. In this report, we have tested the effect of *in utero* and early life exposure to DE versus adult exposure to DE on susceptibility to heart failure in adult mice.

## Results

### In utero and early life or adult exposure to DE does not affect baseline cardiac function

**Figure 1:**
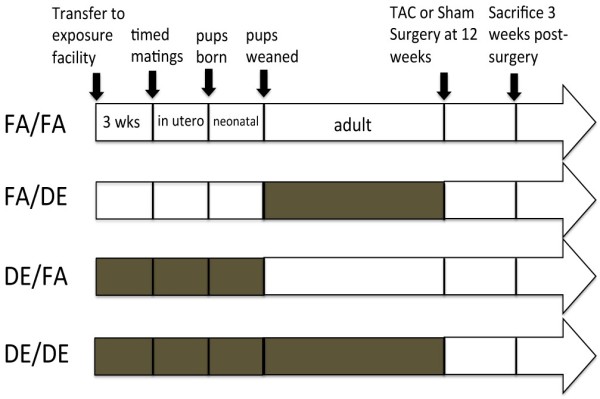
**Schematic diagram indicating diesel exhaust (DE) exposure paradigm.** Four exposure groups tested the effect of developmental or adult exposure to DE or filtered air (FA) exposures on adult cardiac function. Brown color in horizontal arrows represents DE exposure (300 μg/m^3^, 6 hours per day, 5 days per week), while white color represents FA exposure.

**Figure 2:**
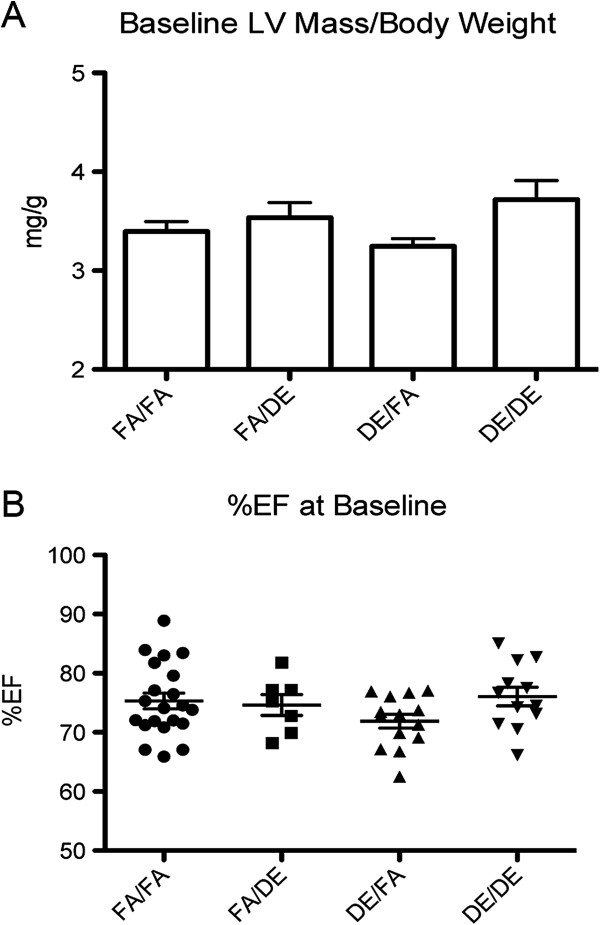
**Echocardiographic assessment of cardiac hypertrophy and function at 12 week of age, prior to TAC/Sham surgeries. (A)** Calculated LV Mass normalized to body weight and **(B)** percentage ejection fraction (%EF) in FA/FA (n = 21), FA/DE (n = 7), DE/FA (n = 14), and DE/DE (n = 12) groups.

To assess the effect of developmental exposure to DE, mice were exposed to filtered air (FA) or DE in four different paradigms as shown (Figure 1) and as described in Materials and Methods. To characterize the effect of these DE exposures on adult cardiac function, we performed an echocardiographic assessment on all male mice at 12 weeks of age. We did not observe either developmental, adult, or a combined exposure to DE to have any significant effect on hypertrophy, as measured by calculated left ventricular (LV) mass (Figure 2A), or cardiac function, as measured by percentage ejection fraction (%EF) (Figure 2B).

### In utero and early life exposure to DE exacerbates the response to pressure overload-induced heart failure

**Figure 3:**
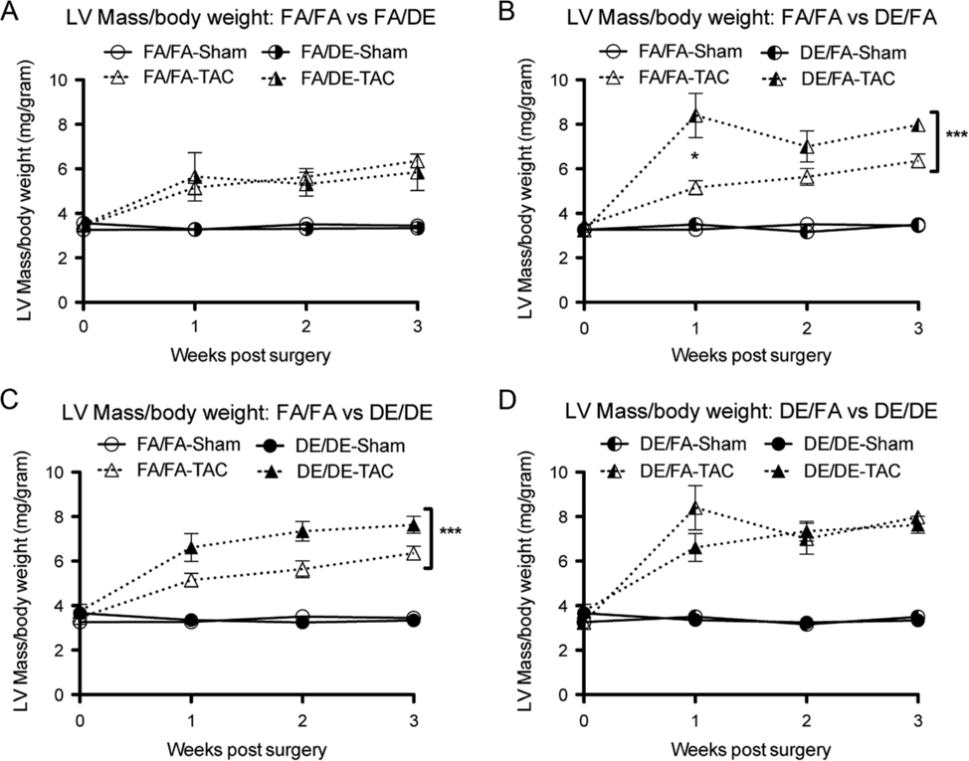
**Echocardiographic assessment of calculated LV Mass normalized to body weight over time following transverse aortic constriction (TAC) or sham surgeries. (A)** FA/FA (n = 7 sham, n = 8 TAC) vs. FA/DE (n = 3 sham, n = 3 TAC), **(B)** FA/FA vs. DE/FA (n = 5 sham, n = 5 TAC), **(C)** FA/FA vs. DE/DE (n = 5 sham, n = 4 TAC) groups, and **(D)** DE/FA vs. DE/DE.

**Figure 4:**
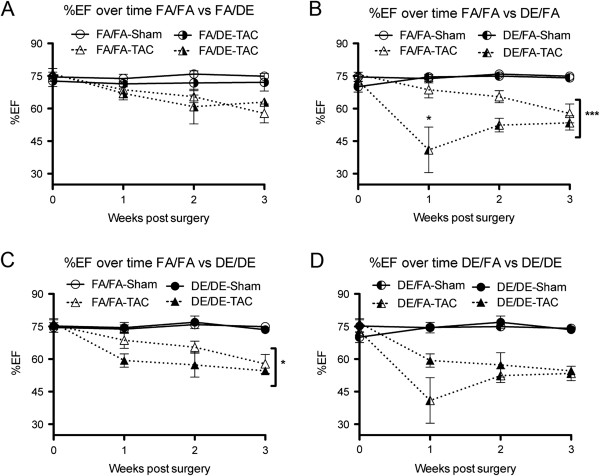
**Percentage ejection fraction (%EF) determined by echocardiography over time following transverse aortic constriction (TAC) or sham surgeries. (A)** FA/FA (n = 7 sham, n = 8 TAC) vs. FA/DE (n = 3 sham, n = 3 TAC), **(B)** FA/FA vs. DE/FA (n = 5 sham, n = 5 TAC), **(C)** FA/FA vs. DE/DE (n = 5 sham, n = 4 TAC) groups, and **(D)** DE/FA vs. DE/DE.

Although our baseline echocardiographic assessment did not reveal any significant differences, we tested the effect of these exposures on pressure overload-induced heart failure by use of the transverse aortic constriction (TAC) model. We observed *in utero* and early life exposure to DE to strongly accelerate the progression of TAC-induced cardiac hypertrophy and systolic dysfunction as measured by increased calculated LV Mass (Figure 3B and C) and reduced%EF (Figure 4B and C). Interestingly, we did not observe any effect of adult exposure to DE on susceptibility to TAC-induced heart failure (Figures 3A and 4A). In assessing the effect of adult exposure to DE on susceptibility to heart failure, FA/DE mice responded in a nearly identical manner to the FA/FA controls (Figures 3A and 4A). Interestingly, we did not observe DE/DE mice to have a worse phenotype than observed in the DE/FA mice, but rather there were trends to suggest DE/DE mice were less susceptible to TAC-induced heart failure than the DE/FA group (Figures 3D and 4D). In our DE/FA group, 2 of 5 TAC mice had progressed into such severe heart failure at 1-week post surgery that our predetermined criteria for early sacrifice was met. No other mouse in any treatment group reached our early sacrifice criteria, potentially suggesting DE/FA mice to be particularly sensitive to TAC-induced heart failure.

**Figure 5:**
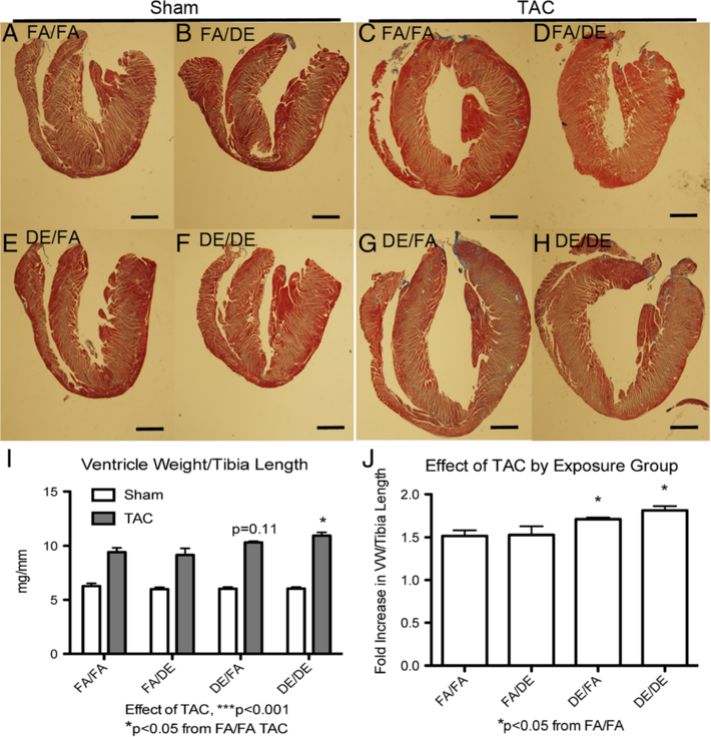
**Gravimetric analysis of ventricle weight at necropsy.** Representative images of sagittal sections of hearts from mice three weeks post sham or transverse aortic constriction (TAC) surgeries **(A-H)**, scale bar = 1 mm. Gravimetric analysis of ventricles normalized to tibia length (VW/tibia length) in FA/FA (n = 7 sham, n = 8 TAC), FA/DE (n = 3 sham, n = 3 TAC), DE/FA (n = 5 sham, n = 5 TAC) and DE/DE (n = 5 sham, n = 4 TAC) groups **(I)**. Effect of TAC by exposure group was also represented as a fold effect of TAC on VW/tibia length normalized to sham mice **(J)**.

Following necropsy, ventricles were removed and assessed gravimetrically (Figure 5). We observed TAC-induced heart failure to increase the ventricle weight normalized to tibia length of all treatment groups (Figure 5I). We observed *in utero* and early life exposure to DE, in combination with adult exposure to DE (DE/DE) to result in significantly elevated ventricle weights in comparison to FA mice. *In utero* and early life exposed mice (DE/FA) showed a trend of increased ventricle weights normalized to tibia length (p = 0.11), but significance was not achieved. When the effect of TAC on increased ventricle weight is normalized to sham controls, we observe DE/FA and DE/DE mice to have a response to TAC that is significantly greater than FA/FA (Figure 5J). In accordance with our echocardiography data, we did not observe adult exposure to DE alone (FA/DE) to have any effect on ventricle weights (Figure 5I and J).

### In utero and early life exposure to DE promotes cardiac fibrosis in pressure overload-induced heart failure

**Figure 6:**
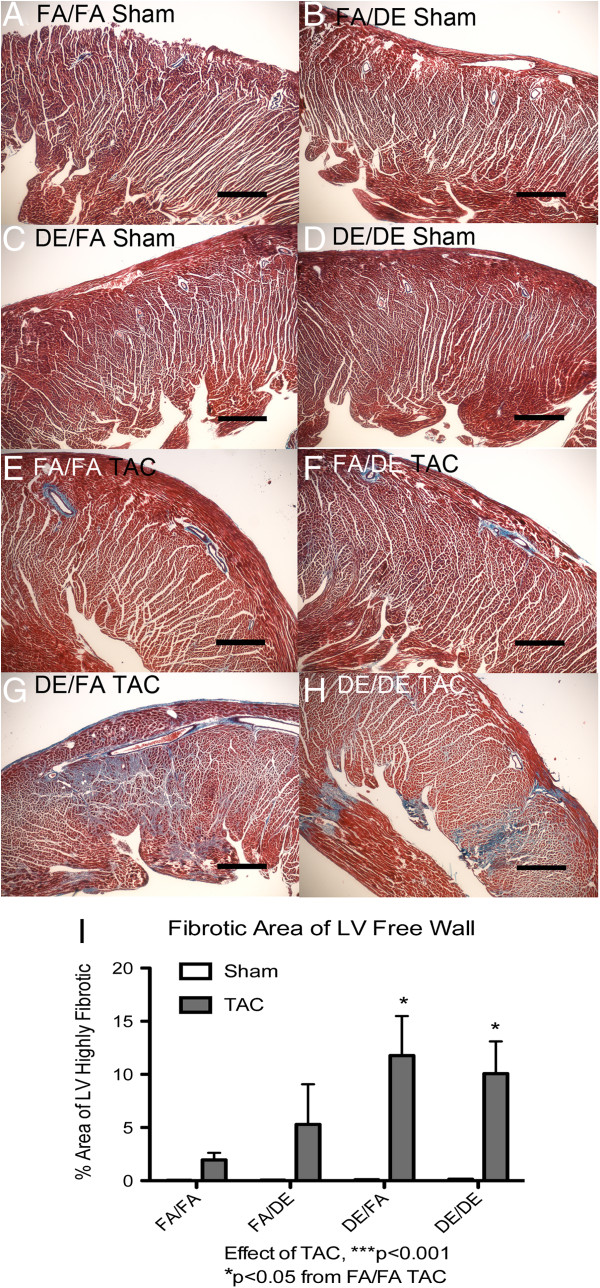
**Assessment of cardiac fibrosis by Masson’s Trichrome staining.** Representative images (4X objective) of sagittal sections of left ventricle free wall from mice three weeks post sham or transverse aortic constriction (TAC) surgeries **(A-H)**, scale bar = 0.5 mm. Fibrotic regions of cardiac tissues are determined by blue staining in FA/FA (n = 5 sham, n = 5 TAC), FA/DE (n = 3 sham, n = 3 TAC), DE/FA (n = 5 sham, n = 5 TAC) and DE/DE (n = 5 sham, n = 4 TAC) groups. Assessment of percentage highly fibrotic regions was quantified **(I)**.

To assess the potential effect of DE on development of cardiac fibrosis after pressure overload, we measured the extent of fibrosis within the myocardium by Masson’s Trichrome staining. In sham-operated mice, we did not observe either developmental, adult, or combined exposure to DE to have any effect on cardiac fibrosis (Figure 6A-D). In mice that underwent TAC, we observed an increase in myocardial fibrosis in all exposure groups (Figure 6E-I) compared to sham-operated controls. Interestingly, we observed an increased fibrotic response in the *in utero* and early life DE exposure cohort (DE/FA) in comparison to FA controls (Figure 6E-I). In FA/FA mice, we observed TAC-induced heart failure to result in largely perivascular fibrosis that is limited in extent (Figure 6E), whereas *in utero* and early life exposed mice reveal extensive interstitial fibrosis that is not limited to a perivascular distribution (Figure 6G and H). DE/DE mice also showed extensive myocardial fibrosis that exceeded that found in FA/FA or FA/DE mice (Figure 6H). We observed a trend where DE/DE mice appeared to have less extensive myocardial fibrosis than that observed in our *in utero* and early life exposed group (DE/FA), but this trend did not reach significance.

### In utero and early life exposure to DE does not increase individual cardiomyocyte cross sectional area

**Figure 7:**
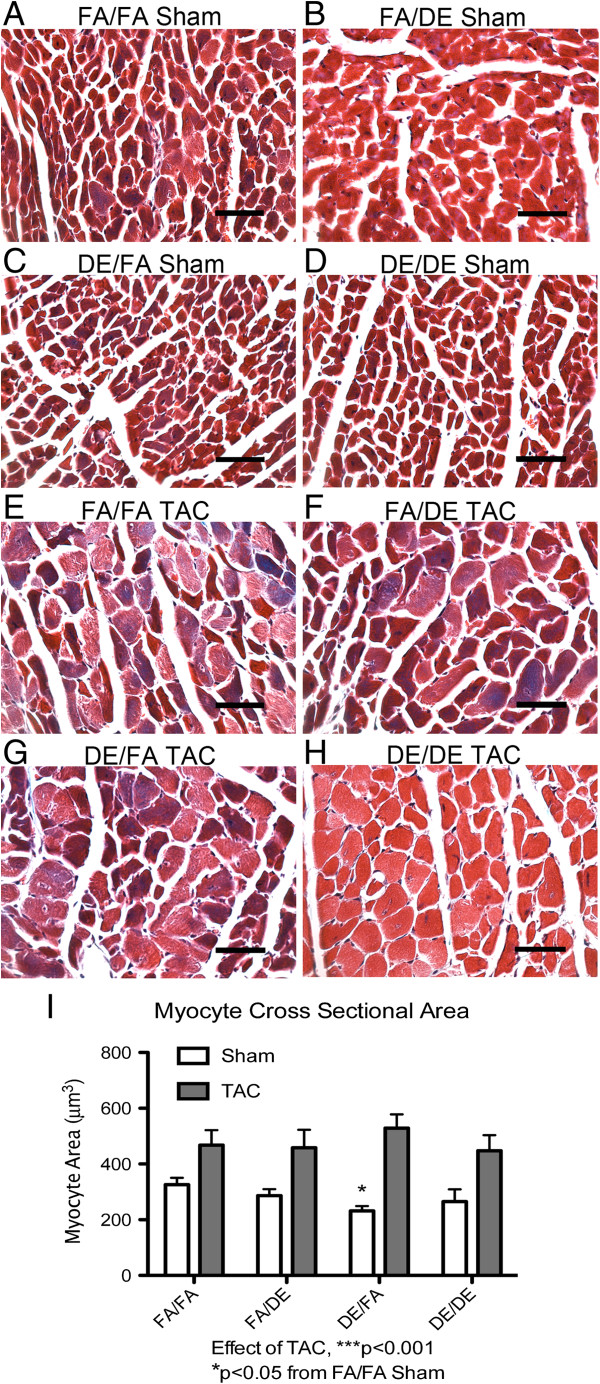
**Measurement of individual cardiomyocyte hypertrophy.** Representative images (40X objective) of sagittal sections of left ventricle free wall from mice three weeks post sham or transverse aortic constriction (TAC) surgeries **(A-H)**, scale bar = 50 μm. Individual cardiomyocyte areas were traced, quantified, and analyzed in FA/FA (n = 5 sham, n = 5 TAC), FA/DE (n = 3 sham, n = 3 TAC), DE/FA (n = 5 sham, n = 5 TAC) and DE/DE (n = 5 sham, n = 4 TAC) groups **(I)**.

As *in utero* and early life exposure to DE appears to accelerate TAC-induced cardiac hypertrophy and increase ventricle weight, we measured individual cardiomyocyte area within the left ventricle to determine if individual cardiomyocyte hypertrophy is responsible for these observations. TAC promoted increased individual cardiomyocyte area in all treatment groups (Figure 7), but we did not observe either *in utero* and early life, adult, or combined DE exposure to have any additional effect on individual cardiomyocyte area (Figure 7E-I). Interestingly, *in utero* and early life DE exposure was associated with a significant reduction in individual cardiomyocyte area in sham-operated mice (Figure 7C and I), suggesting a potential baseline atrophic cardiomyopathy. This effect was not observed in sham DE/DE mice, although there was a trend towards a reduced myocyte area (FA/FA Sham vs DE/DE Sham *T*-Test, p = 0.29).

### In utero and early life exposure to DE is associated with increased lung weight after TAC and altered inflammatory cytokine expression

**Figure 8:**
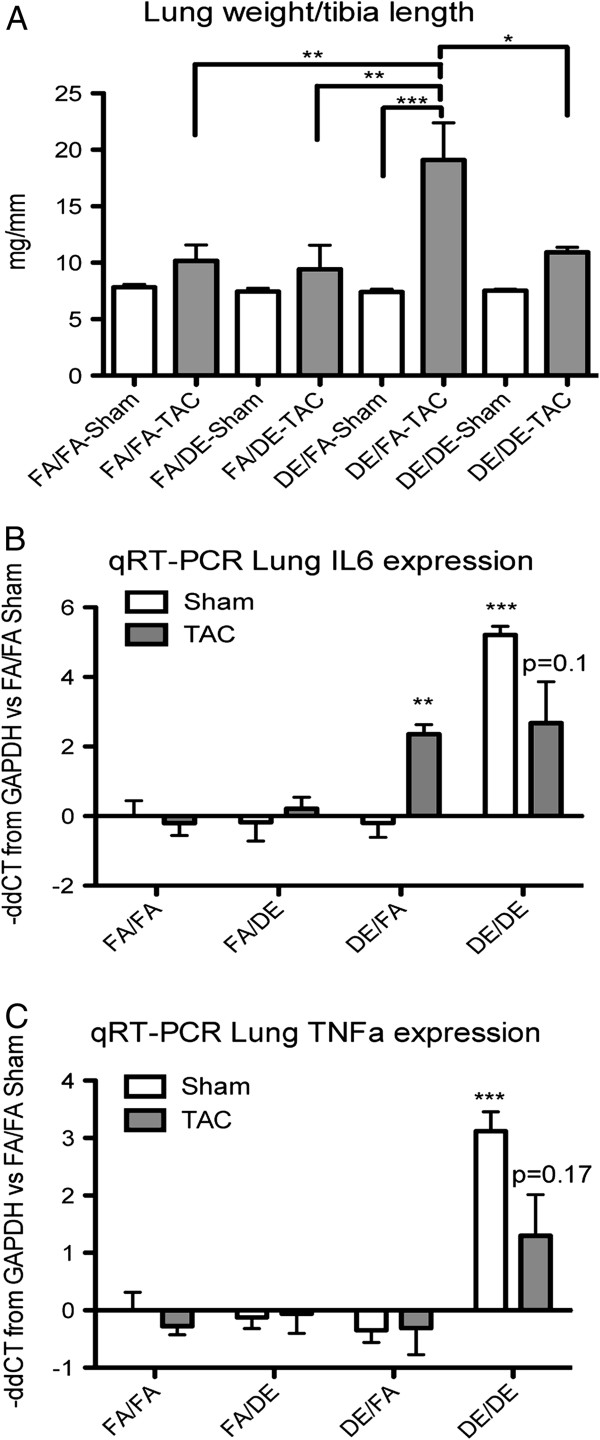
**Effects of DE and TAC on lung weight and inflammatory cytokine expression. (A)** Lung weights as measured at necropsy were collected and analyzed in FA/FA (n = 7 sham, n = 8 TAC), FA/DE (n = 3 sham, n = 3 TAC), DE/FA (n = 5 sham, n = 5 TAC) and DE/DE (n = 5 sham, n = 4 TAC) groups. Relative real-time PCR analysis of IL6 **(B)** and TNFα **(C)** mRNA content in the lung as expressed as negative delta delta CT from FA/FA sham normalized to GAPDH.

To assess the effect of developmental and adult exposure to DE on pulmonary congestion that develops in association with TAC-induced heart failure, lung weights were measured at necropsy and normalized to tibia length. In sham-operated mice, we did not observe *in utero* and early life, adult, or a combined DE exposure to have any effect on lung weights (Figure 8A). In TAC-operated mice, the lung weights of DE/FA TAC mice were significantly greater than all other groups, indicating that *in utero* and early life exposure to DE promotes an exacerbated lung response to TAC (Figure 8A).

As both acute and chronic exposure to DE has been suggested to incite a pulmonary cytokine response, we measured the relative mRNA content of the proinflammatory cytokines interleukin 6 (IL6) and tumor necrosis factor alpha (TNFα) in the lung tissue from all sham and TAC groups. In FA/FA control mice, we did not observe the induction of hypertrophy by TAC to alter IL6 or TNFα mRNA content in comparison to FA/FA sham mice (Figure 8B and C). We also did not observe adult exposure to DE in the absence of developmental DE exposure (FA/DE) to have any effect on lung IL6 or TNFα mRNA content in either sham or TAC mice. Interestingly, although *in utero* and early life DE exposure (DE/FA) did not alter IL6 or TNFα mRNA content in sham mice, we did observe IL6 to significantly increase in DE/FA TAC mice (−ddCT = 2.358, ~5-fold increase). Unexpectedly, we observed *in utero* and early life DE in addition to adult DE exposed mice (DE/DE) to have a dramatic increase in both IL6 and TNFα mRNA content in sham mice (−ddCT IL6 = 5.213, ~37-fold increase, -ddCT TNFα = 3.117, ~8-fold increase). We observed DE/DE TAC mice to have a trend toward reduced inflammatory cytokine expression in comparison to DE/DE sham mice, indicating that the inflammatory cytokine expression is independent of TAC-induced heart failure.

## Discussion

In the present study, we investigated the effect of *in utero* and early life exposure to diesel exhaust (DE) air pollution (≈300 μg/m^3^ PM_2.5_, 6 hrs/day, 5 days/week) on adult susceptibility to pressure overload-induced heart failure in male mice. This concentration of DE was chosen as it is comparable to other studies investigating the vascular effects of DE inhalation [7,9,28] and is relevant to population exposures in highly polluted urban environments [29]. Our study focused on male offspring to control for cyclic hormonal variation. Our observation that *in utero* and early life exposure to DE promotes the development of heart failure in adult animals is consistent with the work of Barker and colleagues [17-23] and implies that adult cardiovascular disease may have more origins in developmental exposure to air pollution than is currently appreciated.

The mechanisms by which *in utero* and early life DE exposures promote adult cardiovascular disease are poorly understood. In rodent models, acute and chronic inhalation of PM_2.5_ can elicit systemic vascular inflammation and impair vascular reactivity [8,10]. These mechanisms have been implicated to play an important role in the progression of cardiovascular disease as well as increase the risk of acute myocardial infarction [30-32] in adults. Appropriate vascular function is critical during embryogenesis and is vital in regulating placental blood flow and nutrient transfer to the developing fetus [33,34]. Exposure to DE during pregnancy in mouse models has been shown to promote fetal inflammation and to promote effects on both pulmonary and neuronal function in the adult offspring [26,27,35]. In addition, human exposure to PM_2.5_ during pregnancy has been shown to decrease placental mitochondrial DNA [36], an indicator of oxidative stress, as well as cause placental DNA hypomethylation [37]. Similar observations have been observed with individuals who smoke tobacco during pregnancy, which has long been known to impact placental blood flow and result in intrauterine growth restriction (IUGR) [38-41]. Affected fetuses demonstrate hypoglycemia, hypoinsulinemia, acidosis, hypoxia, and decreased branched chain amino acid transfer resulting in long lasting adverse effects postnatally [42-47]. Rodent models have shown that exposure to uteroplacental insufficiency results in altered mitochondrial β-oxidation within muscle [48], altered fatty acid metabolism [49], increased visceral adipose tissue deposition [50], and decreased insulin sensitivity associated with elevated fasting plasma glucose [51]. These effects are thought to be due to epigenetic ‘reprogramming’, where changes in DNA methylation and histone modification have been observed to promote long lasting effects on gene expression that alter physiological and cellular function [42,52].

Epigenetic linkage between prenatal environmental insults and adult cardiovascular phenotypes has been previously reported. Patterson et al. recently reported that prenatal hypoxia causes hypermethylation of the PKCϵ promoter, conferring susceptibility to ischemic injury to the adult rat heart [53,54]. Our future investigations on the effect of prenatal exposure to air pollution on adult cardiovascular disease will focus on the potential for these exposures to elicit long-term epigenetic modifications.

The current evidence showing an effect of prenatal or early life exposure to air pollution on adult risk of disease in human populations is limited largely to adult pulmonary function [55] as there is clear epidemiological evidence that childhood exposure to air pollution impairs lung development [56]. Pulmonary inflammation has been postulated to promote cardiovascular disease [57]. Our study demonstrates that combined prenatal and early life exposure to air pollution can alter adult pulmonary cytokine expression and promote pressure overload-induce adult heart failure in mice, but such results cannot necessarily be extrapolated to human populations. The effects of prenatal or childhood air pollution exposure on adult cardiovascular risk in human populations are unknown. Nevertheless, we believe our data provides a strong impetus to investigate whether *in utero* and/or early life air pollution exposure affects adult risk of cardiovascular disease in human populations.

In our report, we found that an 8-week exposure to DE in adult mice did not have any effect on cardiac function at baseline or following TAC-induced heart failure, which is consistent with our previous study in which we investigated the effects of prolonged adult DE exposures (up to 6-months) and failed to observe any effect in two mouse models of cardiac hypertrophy [58]. Others have reported that exposure to concentrated ambient particulate matter (CAPs) potentiates the cardiac hypertrophic response to angiotensin II (AngII) stimulation [16] and promotes cardiac fibrosis [15]. We believe that CAPs have unique cardiotoxicity in comparison to DE, as discussed previously [58]. Our findings indicate that, in mice, the developing organism is much more susceptible to the cardiovascular effects of DE than the adult.

## Conclusions

In summary, we have observed that exposure to diesel exhaust air pollution during *in utero* and early life development in mice increases adult susceptibility to pressure overload-induced heart failure. This effect was not observed in mice exposed to diesel exhaust as adults. This report suggests for the first time that developmental exposure to air pollution may be critical in mediating adult susceptibility to heart failure, and provides impetus to perform appropriate clinical studies to determine whether these findings are applicable to human populations.

## Materials and methods

### Diesel exhaust exposure and mice

Male and female C57Bl/6 J mice were purchased from The Jackson Laboratory (Bar Harbor, Maine, USA). All mice were housed in specific pathogen free (SPF) conditions on a 12/12-light/dark cycle. Female and male mice between the ages of 12 to 14 weeks were transferred to our Northlake Diesel Exposure Facility located near the University of Washington (UW) and housed under SPF conditions in Allentown caging systems (Allentown, NJ, USA) as previously described [59-61]. All animal experiments were approved by the UW Institutional Animal Care and Use Committee. Diesel exhaust (DE) was generated from a single cylinder Yanmar diesel engine (Model YDG5500EV-6EI) operating on 82% load. A detailed analysis of DE particulate components in this system has been previously reported [59]. DE exposures were conducted for 6 hours per day (9 am – 3 pm) five days a week (Monday – Friday) and DE concentrations were regulated to ≈ 300 μg/m^3^ of PM_2.5_. A 300 μg/m^3^ concentration of PM_2.5_ six hours/day, five days/week equates to a time weighted hourly average of 53 μg/m^3^. The following exposure characteristics were measured with DE generated under the same conditions within the exposure facility, though not on the days of this experiment. Oxides of nitrogen concentrations were 1800 ppb NO_x_ and 60 ppb NO_2_, measured using a Thermo Scientific Model 42C analyzer. The concentration of carbon monoxide was 2 ppm, measured using a Langan analyzer, Model T15n. The concentration of carbon dioxide was 1000 ppm with infrared detection (Telaire Model 1050, Telaire Systems). The mass fraction of particle-bound polycyclic aromatic hydrocarbons (PAH) was 20 ng/μg PM_2.5_, measured with an Ecochem PAS 2000. The ratio of the organic carbon to elemental carbon mass concentration was 0.10, based on quartz filter samples adjusted with a concurrent dynamic blank; samples were analyzed by Sunset Laboratories using the IMPROVE A thermo optical reflectance method. The mass median aerodynamic diameter was 85 nm (GSD 1.2), which was obtained by gravimetric analysis of samples collected with a micro orifice uniform deposit impactor (MOUDI, MSP Model 110-NR). The count median thermodynamic equivalent diameter was 87 nm (GSD 3.0), measured with a P-Trak Ultrafine Particle Counter, Model 8525.

Female mice were exposed to either FA or DE for 3 weeks, and then paired with male mice for timed matings during weekend FA exposures. After observation of a vaginal plug, pregnant mice were then re-exposed to the same condition (FA or DE) for the duration of gestation and until weaning at postnatal day (PND) 21. At PND21, offspring were transferred to either FA or DE to continue exposure for 8 additional weeks, representing an “adult” exposure. Male mice exposed to FA throughout the study are designated FA/FA, those mice exposed to FA until weaning and subsequently transferred to DE are designated FA/DE, mice exposed to DE until weaning and transferred to FA are designated DE/FA, and mice exposed to DE throughout the study are designated DE/DE.

A total of 9 female mice became pregnant in FA exposures and 8 female mice became pregnant in DE exposures. Of 9 FA litters, 5 remained in FA exposures, while 4 were transferred to DE. Of 8 DE litters, 5 were transferred to FA, and 3 remained in DE. At 11 weeks of age, male offspring were transferred from the Northlake facility to the UW Medicine South Lake Union (SLU) SPF vivarium, where each mouse underwent echocardiographic assessment and surgery. A total of 54 male offspring were used for this study.

### Echocardiography and transverse aortic constriction surgeries

Upon transfer of male offspring to the SLU SPF vivarium, mice underwent baseline echocardiographic assessment at 11–12 weeks of age. Under 0.5% isoflurane anesthesia, cardiac size and function were assessed using a Visual Sonics (Toronto, Canada) VEVO 770 system equipped with a 707B scan head as previously described [62,63]. When the heart rate of the mouse had returned to normal followed anesthesia (>520 bpm), parasternal short axis views were obtained under M-mode. Cine loops collected from M-mode views were analyzed for anterior and posterior LV wall thickness as well as LV internal diameter at diastole and systole. Percentage ejection fraction (%EF) and LV Mass were calculated from Visual Sonics Standard Measurements and Calculations. One week after baseline echocardiographic assessment was completed, male mice were randomly assigned to either transverse aortic constriction (TAC) or sham surgeries. For surgeries, male mice between 12 and 14 weeks of age were anesthetized using ketamine (130 mg/kg i.p.) and xylazine (8.8 mg/kg i.p.) and subjected to transverse aortic constriction using a 27-gauge needle as described [62,63]. To measure cardiac response to surgery, echocardiographic assessments were completed for each mouse at 1 week, 2 weeks, and 3 weeks post surgery. All echocardiographic measurements were performed by a blinded observer. In all exposure groups, we observed 30-40% mortality due to surgical complications, as defined by death within 72 hours of surgery.

### Necropsy and histology

All mice were euthanized at 3 weeks post surgery, or earlier if predetermined euthanasia criteria were met (ejection fraction below 25% with overt heart failure). Mice were sacrificed by overdose with tribromoethanol (650 mg/kg i.p.) followed by exsanguination. Tissues were collected for gravimetric analysis prior to storage in liquid nitrogen or fixation with 4% paraformaldehyde and subsequent storage in 70% ethanol. Tissues used for histology underwent tissue processing and embedding in paraffin. Seven-micron thick cross sections were made of the heart, and Masson’s Trichrome as well as hematoxylin and eosin staining were performed using standard techniques. Extent of myocardial fibrosis was determined by Masson’s Trichrome stain where percentage of blue stain was quantified over total tissue area from 4X images of the left ventricle free wall using NIH Image J (Bethesda, MD, USA) as previously done [62]. Using NIH Image J, low power images of the LV free wall were assessed in a blind fashion. The border of the myocardium was traced manually to get a baseline area, then the area that would be considered ‘highly fibrotic’, where there is clear evidence of fibroblast collagen deposition and blue staining, is manually traced to get a subsequent area. The area of the myocardium that would be ‘highly fibrotic’ is expressed as a ‘percentage highly fibrotic area’. Individual cardiomyocyte area was quantified using NIH Image J, where the areas of 100 cardiomyocytes were quantified and averaged for each section as described previously [62]. Of 100 cardiomyocytes within the LV, area is averaged to get a single value that represents average myocyte area per heart. We then averaged cardiomyocyte area within exposure and surgery groups to assess individual cardiomyocyte hypertrophy.

### Lung RNA isolation and qRT-PCR of IL6 and TNFα

Frozen lung tissue from the inferior right lobe was collected from each mouse, placed in RNA stabilizing solution Trizol (Invitrogen; Carlsbad, CA, USA) and homogenized with Polytron mechanical homogenizer (Kinematics; Bohemia, NY, USA). RNA isolation was carried out using Qiagen RNeasy Kit (Valencia, CA, USA) following the manufacturer’s protocol. Isolated RNA underwent further purification by standard alcohol precipitation and resuspension techniques. One microgram of RNA was reverse transcribed to cDNA using iScript cDNA synthesis kit (Bio-Rad; Hercules, CA, USA) following the manufacturer’s protocol. Quantitative Real-time PCR (qRT-PCR) was carried out using an ABI7500 Fast Real-Time PCR system (Applied Biosystems, Inc.; Foster City, CA, USA) using Power SYBR Green (Applied Biosystems, Inc.; Foster City, CA, USA) following the thermo-cycling settings of of 5 min 95°C to activate Taq DNA polymerase, followed by 40 cycles of 95°C for 15 seconds and 60°C for 60 seconds. Relative mRNA content of IL6 and TNFα were normalized to GAPDH, using the following primers: *Tnf*α, 5′-CCTGTAGCCCACGTCGTAG-3′ (forward) and 5′-GGGAGTAGACAAGGTACAACCC-3′ (reverse), *Il6,* 5′-TAGTCCTTCCTACCCCAATTTCC-3′ (forward) and 5′-TTGGTCCTTAGCCACTCCTTC-3′ (reverse), *Gapdh*, 5′-CTTCCGTGTTCCTACCC-3′ (forward) and 5′-ACCTGGTCCTCAGTGTAGCC-3′ (reverse). Relative mRNA content of each gene was calculated using the 2(−ddC(T) method as previously described [64] and represented as the change in delta CT between GAPDH and IL6 or TNFα from FA/FA sham to treatment group (−ddCT).

### Statistical analysis

Statistical analyses were performed using GraphPad Prism 5 for Microsoft OS X (GraphPad Software, Inc.; San Diego, CA, USA). Differences between two groups were determined by Student’s *T*-Test using an α-value of 0.05. When more than two groups were being compared, a one-way ANOVA followed by a Tukey’s Posthoc test was used. When statistical analyses between two groups were made following a variable over time, a repeated measure two-way ANOVA was used. In the DE/FA group, after week 1 data was collected, 2 mice met our predetermined early sacrifice criteria and were sacrificed prior to the 3-week time point. To assess significance between DE/FA and FA/FA or DE/DE, we performed a repeated measure two-way ANOVA across all 3 weeks while excluding these two data points. An additional one-way ANOVA followed by a Tukey’s posthoc test was performed at the one-week time point with all data points to confirm significance. All error bars in figures represent mean ± standard error of the mean; *, **, *** represent significant differences with p < 0.05, p < 0.01, and p < 0.001 respectively.

## Abbreviations

PM: Particulate matter; PM2.5: Particulate matter with a diameter of 2.5 μm or less; DE: Diesel exhaust; FA: Filtered air; TAC: Transverse aortic constriction; LV: Left ventricle; EF: Ejection fraction; bpm: beats per minute; TNFα: Tumor necrosis factor alpha; IL6: Interleukin 6; GAPDH: Glyceraldehye 3-phosphate dehydrogenase; qRT-PCR: Quantitative real time polymerase chain reaction; -ddCT: Negative delta delta threshold cycle; CAPs: Concentrated ambient particulate matter; SPF: Specific pathogen free; i.p.: intraperitoneal injection; cDNA: complimentary deoxyribonucleic acid; mRNA: messenger ribonucleic acid; ANOVA: Analysis of variance.

## Competing interests

The authors have no conflicts to disclose.

## Authors’ contributions

CSW, contributed to generating hypothesis and experimental design, conducted experiments, interpreted data, and is the primary author of the manuscript; YL, performed animal surgeries, contributed to preparation of manuscript; YCC, performed qRT-PCR experiments, contributed to preparation of manuscript; IOM, analyzed echocardiography cine loops, contributed to preparation of manuscript; JRF, performed diesel exhaust characterization experiments, contributed to preparation of manuscript; TVL, contributed to diesel exhaust characterization experiments, contributed to preparation of manuscript; WMC, assisted in animal necropsies, contributed to preparation of manuscript; MTC, contributed to generating hypothesis and experimental design, contributed to preparation of manuscript and was responsible for overall coordination of the project. All authors read and approved of the final copy of the manuscript.

## Authors’ information

CSW is a postdoctoral fellow and toxicologist. YL is a junior faculty member and cardiovascular scientist. YCC is a graduate student in toxicology. IOM is a clinical cardiology fellow. JRF is a postdoctoral fellow and exposure scientist. TVL is a senior faculty member, atmospheric scientist and environmental engineer. WMC is a staff research scientist. MTC is a senior faculty member, cardiovascular scientist and practicing cardiologist.
